# A case of ruptured hepatic metastases during pembrolizumab administration for cutaneous malignant melanoma

**DOI:** 10.1016/j.abd.2022.10.015

**Published:** 2024-02-01

**Authors:** Shohei Igari, Miyuki Yamamoto, Nobuyuki Kikuchi, Mikio Ohtsuka, Toshiyuki Yamamoto

**Affiliations:** Department of Dermatology, Fukushima Medical University, Fukushima, Japan

Dear Editor,

A 76-year-old female visited our department complaining of pigmented lesions on the right heel that had appeared 10 years previously (Fig. 1). She was diagnosed with cutaneous acral lentiginous melanoma and underwent resection of the lesions. Histological examination revealed atypical cells infiltrating irregularly from the dermis (Fig. 2). These atypical cells were immunoreactive for MART-1 and HMB-45 (Breslow Depth: 3.7 mm, T3bN0M0). Computed Tomography (CT) scan at 18 months of follow-up revealed multiple liver and lung metastases. Pembrolizumab administration was started, and the hepatic metastases were gradually reduced in size. On the day of the 21st dose of pembrolizumab, the patient was pale and complained of nausea and sharp pain in the upper abdomen. The complete blood count showed severe anemia (red blood cells 216×10^4^/µL; hemoglobin 6.6 g/dL). Abdominal CT scan demonstrated a massive hemoperitoneum with arterial bleeding, suggesting a rupture of the metastatic lesions in the left hepatic lobe (Fig. 3). Transcatheter arterial embolization (TAE) of the left hepatic artery with gelatin sponge particles was immediately performed. By the emergency TAE, intraperitoneal bleeding was temporarily stopped, but abdominal CT on the 25th day after TAE indicated that the hematoma in the liver was significantly increased in size. Since arterial rebleeding was not detected, the hematoma growth was considered to be caused by oozing from necrotic metastatic lesions. The patient’s medical condition rapidly deteriorated, and she was transferred to another hospital for palliative care.

**Figure 1:**
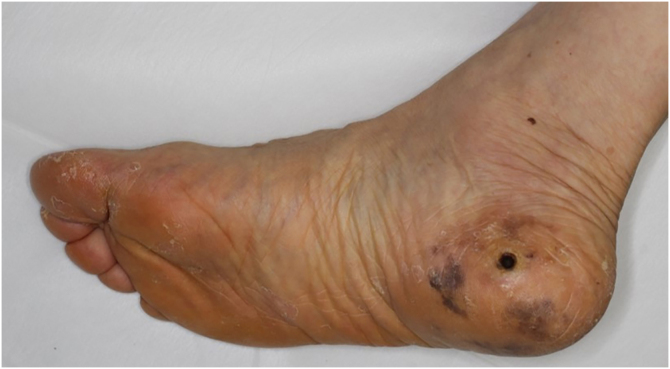
Clinical presentation. Multiple pigmentation patches on the heel.

**Figure 2:**
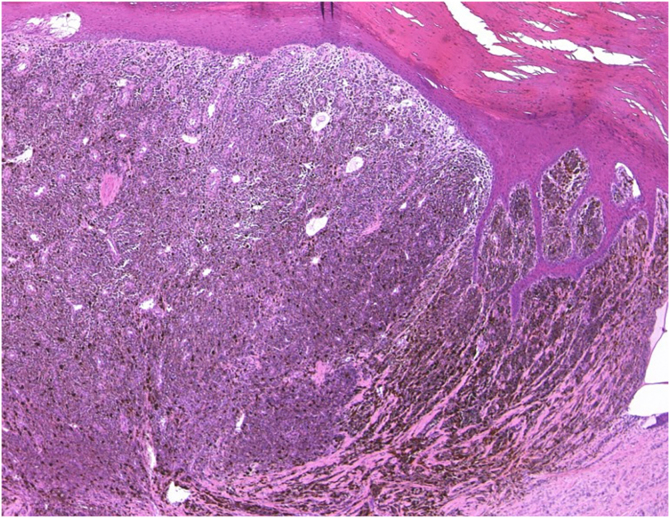
Histological feature showing proliferation of atypical melanocytes in the dermis (×100).

**Figure 3:**
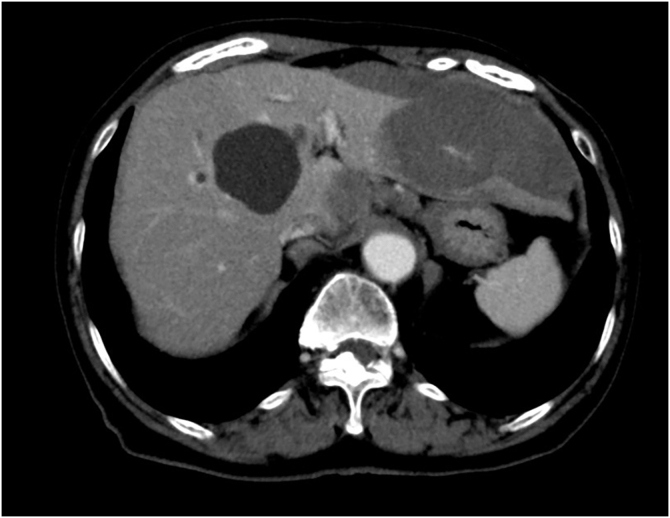
Abdominal CT showed a rupture of the liver metastases.

With regard to cutaneous melanoma, only 9 cases of ruptured hepatic metastases have been reported in the literature.^1^ Among them, 5 patients received resection of the hepatic lobes with hemorrhaging metastatic lesions, and the remaining four patients and the currently reported patients were treated with TAE.^1^, ^2^, ^3^, ^4^

At present, TAE is widely used as an initial treatment for intraperitoneal bleeding from ruptured hepatic tumors. However, recent reports have shown that rebleeding occurred following TAE and that the long-term outcome of patients treated with TAE was poor compared to those who received hepatic lobectomy.^5^ In our patient, TAE was temporarily effective in controlling arterial bleeding from the hepatic metastatic lesions, but extravasation from the tumor might have continued and resulted in the enlargement of the intraperitoneal hematoma and aggravation of the physical condition.

To date, there are few reports of ruptured hepatic metastases in melanoma patients treated with immune checkpoint inhibitors. In our patient, the liver metastases responded well to pembrolizumab therapy and dramatically decreased in size at 13 months after starting pembrolizumab. But at after 3 months, rupture of the hepatic metastases occurred, suggesting the possibility that the hepatic metastases rapidly enlarged during these 3 months. The previously reported in most patients died of melanoma progression or rebleeding within a few months after the onset of rupture.

As rupture of hepatic metastasis of malignant melanoma is very rare, the correct diagnosis may be delayed, resulting in rapid fatal outcomes for affected patients. Therefore, physicians should keep such a rare event in mind when treating patients with hepatic metastases of melanoma showing rapidly progressive anemia and abdominal pain.

## Financial support

None declared.

## Authors’ contributions

Shohei Igari: Designed the study, performed the research and contributed to the analysis and interpretation of data, wrote the initial draft of the manuscript, and read and approved the final version of the manuscript.

Toshiyuki Yamamoto: Designed the study, and assisted in the preparation of the manuscript.

Miyuki Yamamoto: performed the research and contributed to the analysis and interpretation of data, read, and approved the final version of the manuscript.

Nobuyuki Kikuchi: Performed the research and contributed to the analysis and interpretation of data, read, and approved the final version of the manuscript.

Mikio Ohtsuka: Performed the research and contributed to the analysis and interpretation of data, assisted in the preparation of the manuscript, read, and approved the final version of the manuscript.

## Conflicts of interest

None declared.
